# Morphological aspects of immature stages of *Migonemyia migonei* (Diptera: Psychodidae, Phlebotominae), an important vector of Leishmaniosis in South America, described by scanning electron microscopy

**DOI:** 10.1371/journal.pone.0242163

**Published:** 2020-11-12

**Authors:** Eric Fabrício Marialva, Nágila F. Secundino, Fernando F. Fernandes, Helena R. C. Araújo, Claudia M. Ríos-Velásquez, Paulo F. P. Pimenta, Felipe A. C. Pessoa

**Affiliations:** 1 Laboratório de Ecologia de Doenças Transmissíveis na Amazônia, Instituto Leônidas & Maria Deane - Fiocruz Amazônia, Manaus, Amazonas, Brasil; 2 Programa de Pós Graduação em Biologia da Interação Patógeno Hospedeiro, Instituto Leônidas & Maria Deane - Fiocruz Amazônia, Manaus, Amazonas, Brasil; 3 Laboratório de Entomologia Médica, Instituto René Rachou - Fiocruz Minas Gerais, Belo Horizonte, Minas Gerais, Brasil; 4 Divisão de Entomologia, Universidade Federal de Goiás, Goiânia, Goiás, Brasil; 5 Laboratório de Biotecnologia Industrial, Instituto de Pesquisas Tecnológicas do Estado de São Paulo (IPT), São Paulo, Brasil; Universidade Federal de Lavras, BRAZIL

## Abstract

We describe the immature stages of *Migonemyia migonei*, which is the vector of *Leishmania (Viannia) braziliensis*, the etiological agent of cutaneous leishmaniasis in South America, and a putative vector of *Leishmania infantum chagasi*. Scanning Electron Microscopy (SEM) was used to refine the description of the structures of the egg, all instar larvae, and the pupa. The eggs have polygonal cells on the egg exochorion, and differences between larval and pupal chaetotaxy have been highlighted. Different sensillary subtypes—trichoidea, basiconica, coelonica and campanoformia—were observed in the larval stages. The results presented herein contribute to the taxonomy of *Mg*. *migonei* and may contribute to future studies on the phylogeny of this important vector species.

## Introduction

In the last few decades, new proposals in sand fly phylogeny have been based primarily on adult morphology, and several authors have adopted the Galati [1, 2] proposal, which changed the taxonomy, phylogeny and nomenclature basis of phlebotomine systematics. However, knowledge of the eggs, larvae and pupae of phlebotomine sand flies (Diptera: Psychodidae) remains incomplete, due to the difficulty of finding natural breeding sites and the difficulties of lab colonization. As a result, out of more than 537 species described in the Neotropics [1–3], only 92 species have been described, or partially described, in their larval stages. Yet, larval structures provide significant information about the taxonomy, phylogeny, and evolution of this subfamily.

Analyzing the microstructure of the immature stages of Phlebotominae reveals morphological characters that can be used to refine taxonomic and phylogenetic studies and better elucidate the evolution of this subfamily. Microstructure analysis also makes it possible to investigate the existence of sense structures that these vectors use for communication, which may aid the development of alternative, eco-friendly control strategies.

The use of scanning electron microscopy (SEM) has significantly improved the characterization and description of immature forms, and provided details of larval chaetotaxy [4, 5]; ontogeny [6–8]; spiracles [9, 10]; and antennal and mouth parts, such as the sensilla and caudal bristles [7, 11, 12]. Despite this, very few studies have analyzed pupal morphology of New World phlebotomine sand flies [8, 13–16], and thus descriptions of immature sand fly forms remain scarce.

The sand fly *Migonemyia*. *migonei* (França) is an important vector of *Leishmania (Viannia) braziliensis*, and one of the causative agents of cutaneous leishmaniasis in South America, especially in Brazil [17–19]. Torrellas et al. [20] found *Mg*. *migonei* infected with *Le*. *(V*.*) guyanensis* and *Le*. *(Leishmania) mexicana* in an Andean region of Venezuela. Other studies have confirmed that this species is associated with the transmission of *Le*. *infantum chagasi* in Brazil and Argentina [21, 22].

Despite the importance of immature morphology, the few studies of *Mg*. *migonei* that have been conducted under scanning electron microscopy (SEM) have been restricted to descriptions of the larvae antennae [11], spiracles [9, 10] and egg exochorion [23, 24].

The present study aims to provide a complete morphological analysis of the surface of immature stages of *Mg*. *migonei* in order to define taxonomic characters that will support future work on phylogenetics and systematics.

## Material and methods

The eggs, larvae, and pupae of *Mg*. *migonei* were acquired from a stable laboratory colony, reared from specimens obtained in the municipality of Baturité, Ceará State, Brazil. The species was bred at the laboratory facilities of Leônidas & Maria Deane Institute, Manaus, Amazonas State, following the method described by Lawyer et al. [25]. Larvae from each larval instar (1^st^ to 4^th^) and pupae were slide-mounted in Berlese fluid. Measurements of the body’s bristles were made under light microscopy.

Morphology and chaetotaxy of the head were observed following the methodology of Arrivillaga et al. [26], which assigns taxonomical importance to the morphology and setae of the mouthparts. Chaetotaxy of the body followed the system used by Ward [27]. Chaetotaxy of the pupae followed the terminology proposed by Oca-Aguilar [8]. Systematic classification followed Galati [1, 2], and generic abbreviations followed Marcondes [28]. In addition, the species has been studied and photographed under scanning electron microscopy. Reared immatures were killed in hot water (70°C), fixed in 3% glutaraldehyde and washed thoroughly in phosphate-buffered saline; the saline solution was changed every 30 min over a period of six hours. Immatures were then fixed in osmium tetroxide, dehydrated in a series of ethyl alcohol concentrations, submitted to critical point drying in carbon dioxide, and spattered with 25 MA colloidal gold [7, 11]. The specimens were examined in a scanning electron microscope (JSM5600, JEOL, Tokyo, Japan) at an accelerating voltage of 7 KV and then photographed. Tables were created to compare the differences in chaetotaxy between the instars, and stages of *Mg*. *migonei*.

## Results

### Egg of *Mg*. *migonei*

**Fig 1:**
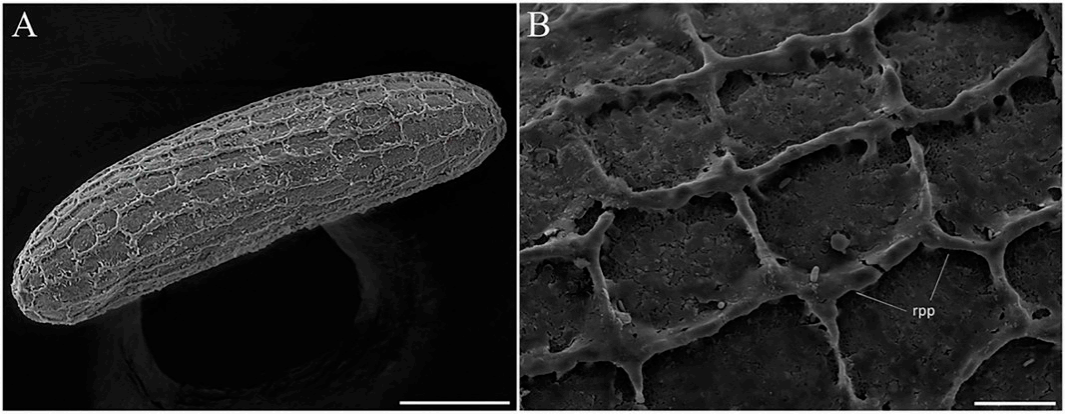
Scanning electron microscopy of the eggs of *Migonemyia migonei*. (A) general view of the egg showing an ornamentation characterized by the presence of ridges arranged in a polygonal pattern (Scale bar: 50 μm). (B) eggshell ornamentation showing detail of the ridges (Scale bar: 10 μm), rpp—ridges polygonal pattern.

The egg is elongated, with one side slightly flattened, measuring 323 (300–351) μm in length and 94.8 (89–107) μm in width (N = 4) (Fig 1A). The exochorion is formed by a thin basal lamina ornamented with polygonal reticulation composed of ridges that are usually continuous and form alternating transversal rows of polygonal cells that are generally rectangular (Fig 1B).

### General appearance of the larvae of *Mg*. *migonei*

The larva is caterpillar-like, with a well-sclerotized hypognathous, non-retractile head and very short antennae with short basal tubercles. Head and body tegument are dark brownish in color and covered by a scattering of very small spines and tubercles. The thorax includes a prothorax with the anterior spiracle borne laterally, and two other segments (meso and metathorax). The abdomen is nine-segmented, covered by brown pale setae and the body tegument is yellowish, with a pair of posterior spiracles borne laterally on a short tubercle.

**Fig 2:**
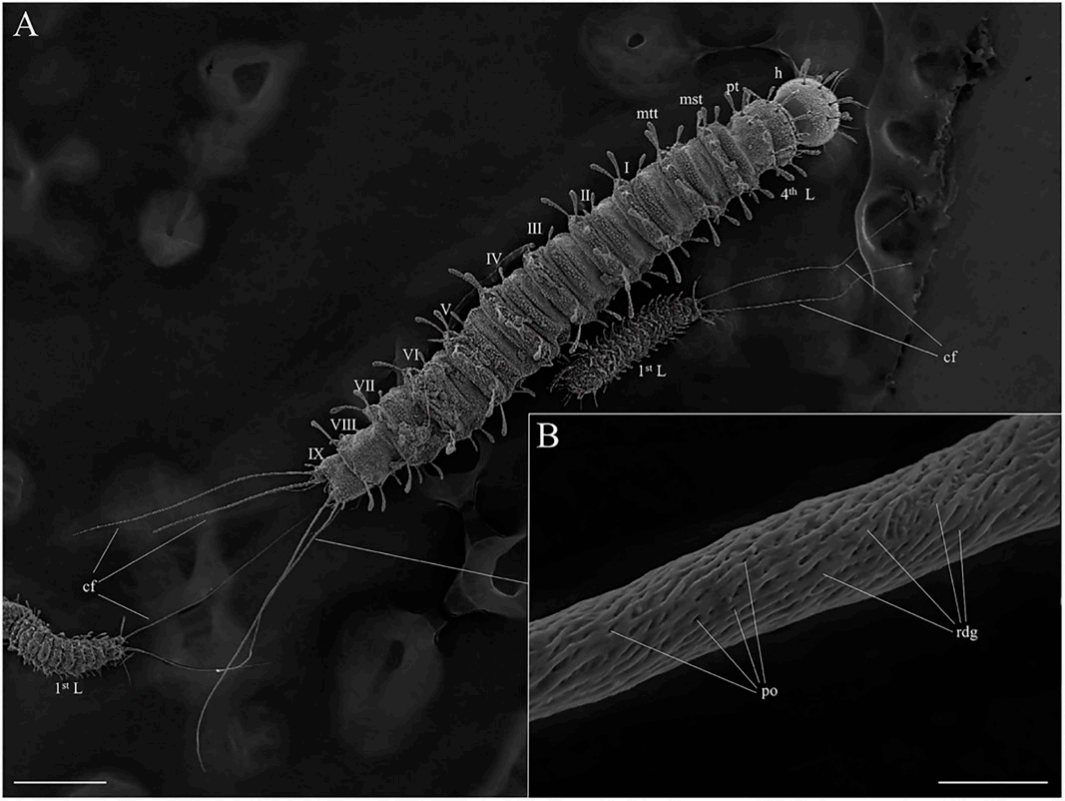
Scanning electron microscopy of the fourth and first instar larvae of *Migonemyia migonei*. (A) observation of first (1^st^) and fourth (4^th^) instar larvae, showing the number of body segments and caudal filaments, dorsal view, cf—caudal filaments; h—head; pt—prothorax; mst—mesothorax; mtt—metathorax (Scale bar: 200μm). (B) higher magnification of the surface of the caudal filament showing pores implanted between non-parallel, interconnected and overlapping ridges, rdg—ridges; po—pores (Scale bar: 5μm).

Caudal filaments or caudal setae: long trichoid sensilla implanted between non-parallel ridges that interconnect (or overlap), darkened, exhibiting many wall pores, and observed to be double-paired in the last three instars, or simply paired in the first instar (Fig 2A and 2B).

**Fig 3:**
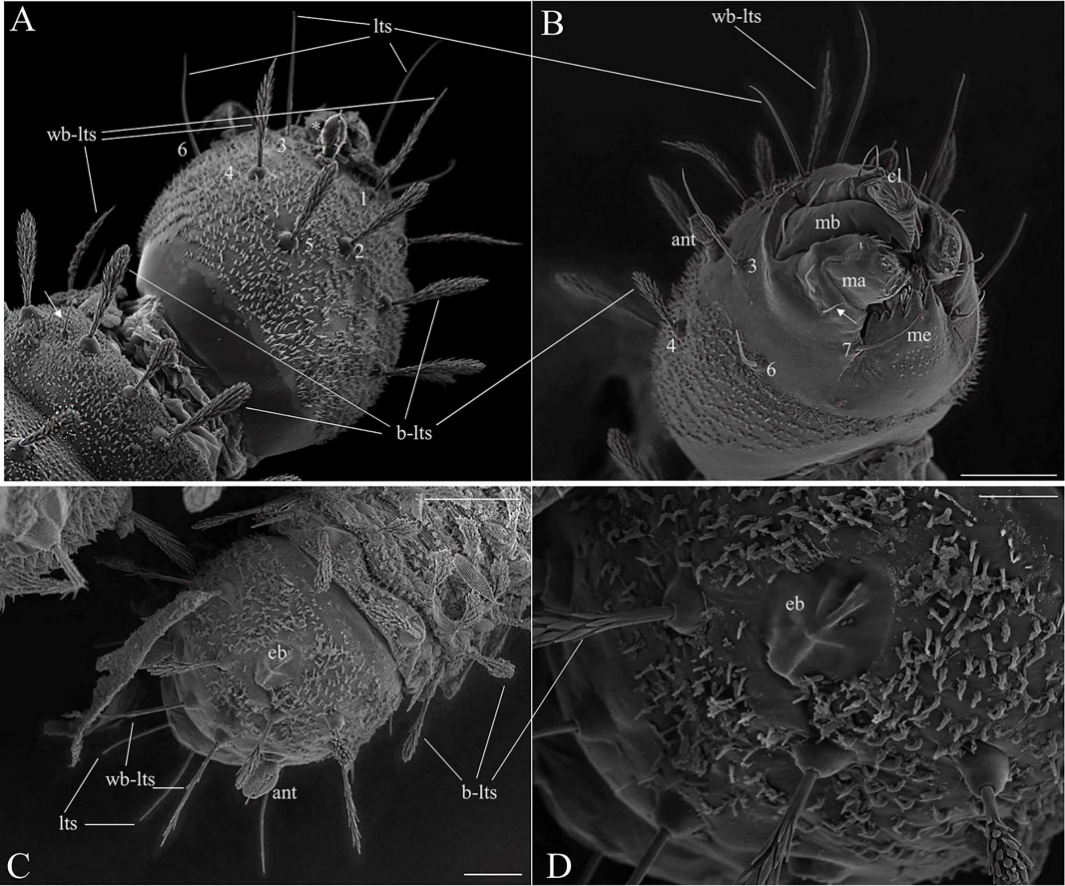
Scanning electron microscopy of the larva of *Migonemyia migonei*. (A) Head of the fourth instar larva in dorsal and ventral view. (B) Long trichoid sensilla observed at the apex of the head (lts) and short trichoid sensilla on the mouthparts (arrow) (A and B scale bars: 20 μm). (C) Head of first instar in dorsal view. Forehead exhibits two weakly brush-like trichoid sensilla (wb-lts) inserted between and slightly forward of the antennae (ant), and long trichoid sensilla (lts) that are inserted further down toward the mouthparts (Scale bar: 20 μm). (D) eb—egg buster; cl—clipeo; md—mandible; ma—maxilla; m—mentum. The setae were numbered according to the chaetotaxy proposed herein: (1 = wb-lts) frontoclipeal anterior setae (weakly brush-like trichoid sensilla); (2 = b-lts) frontoclipeal posterior setae with barbed shape (brush-like trichoid sensilla); (3 = lts) the genal anterior setae with a simple spine form (long and bare trichoid sensilla); (4) the genal medial and (5) genal posterior setae are barbed and brush-like (4 and 5 = brush-like trichoid sensilla); and, in the ventral part (B), (6) the postgenal and (7) subgenal setae are simple (6 and 7 = bare trichoid sensilla).

The head is dark brown (Figs 2A and 3A). The body is pale with darkened eighth and ninth abdominal segments, tiny spines borne on all segments (Figs 2A and 7), and a prominent egg buster (Fig 3C and 3D) with a peculiar shape is present in the first instar. There are three types of setae, usually distributed in pairs: barbed brush-like setae (of the brush-like trichoid sensilla type) which are most widely distributed on the larval head and body—Fig 3A, seta 2); slightly barbed setae (weakly brush-like trichoid sensilla—Fig 3A, seta 1); and simple, bare, paired-setae (trichoid sensilla—Fig 3A, seta 6). The size and the type of setae are shown in Tables 1–6.

**Table 1:** Head (setae number, type of setae, size of the setae in μm, N = 4) of the fourth to first instar larvae of *Migonemyia migonei*.

Head	Setae number	Type of setae	Size of the setae in the corresponding larval instar			
		Bristle	4^th^	3^rd^	2^nd^	1^st^
Frontoclipeal anterior	1	Spinulate	93.3	77.4	65.4	52.0
Frontoclipeal posterior	2	Barbed	85.3	65.3	50.7	29.4
Genal anterior	3	Simple	113.0	70.65	60.0	41.4
Genal medial	4	Barbed	93.3	64	53.3	44.0
Genal posterior	5	Barbed	90.7	62.7	46.7	33.4
Postgenal	6	Simple	101.3	64.0	54.7	37.3
Subgenal	7	Simple	80.0	37.3	24.0	13.3

NO: Not observed.

**Table 2:** Prothorax (setae number, type of setae, size of the setae in μm, N = 4) of the fourth to first instar larvae of *Migonemyia migonei*.

Prothorax	Setae number	Type of setae	Size of the setae in the corresponding larval instar			
		Bristle	4^th^	3^rd^	2^nd^	1^st^
Dorsal internal	1	Barbed	85.0	57.3	46.7	38.7
Dorsal intermediate	2	Barbed	65.0	41.35	20.0	24.0
Dorsal external	3	Barbed	87.5	60.0	49.4	NO
"Shoulder" accessory	a	Spine	25.0	17.4	17.7	NO
"Shoulder" accessory	b	Barbed	16.0	08.0	08.0	NO
Anterior ventrolateral	4	Barbed	100.0	61.3	45.4	32.0
Ventral external	5	Barbed	67.5	55.0	49.4	30.7
Ventral internal	6	Barbed	100.0	64	53.3	29.3
Dorsal submedian	7	Barbed	90.0	49.3	33.4	16.0
Mid-dorsal	8	Barbed	100.0	64.0	44.0	18.7
Dorsolateral	9	Barbed	95.0	52.0	40.0	26.7
Basal	10	Barbed	42.5	22.7	12.0	NO
Post-ventrolateral	11	Barbed	96.0	53.35	44.0	30.7
Post-ventral	12	Spine	13.3	6.7	6.7	6.7
Mid ventral	13	Barbed	72.0	42.6	26.7	12.0
Ventral intermediate	14	Barbed	17.3	12.0	NO	2.7
Ventral submedian	15	Barbed	38.7	21.35	13.3	8.0

NO: Not observed.

**Table 3:** Meso and metathorax (setae number, type of setae, size of the setae in μm, N = 4) of the fourth to first instar larvae of *Migonemyia migonei*.

Meso and metathorax	Setae number	Type of setae	Size of the setae in the corresponding larval instar			
		Bristle	4^th^	3^rd^	2^nd^	1^st^
"Shoulder" accessory	a	Spine	14.7	10.7	8.0	6.7
"Shoulder" accessory	b	Barbed	17,3	9,35	8.0	NO
Anterior ventrolateral	4	Barbed	90.0	52.0	37.4	25.4
Dorsal submedian	7	Barbed	142.5	69.35	37.4	20.0
Mid-dorsal	8	Barbed	152.5	78.65	46.7	21.4
Dorsolateral	9	Barbed	127.5	65.3	42.7	29.3
Basal	10	Barbed	25.0	13.3	8.0	NO
Post-ventrolateral	11	Barbed	78.8	49.3	36.0	17.4
Post-ventral	12	Spine	14.7	9.35	NO	05.3
Mid-ventral	13	Barbed	77.3	38.65	29.3	14.7
Ventral intermediate	14	Barbed	22.7	13.3	8.0	4.0
Ventral submedian	15	Barbed	44.0	21.4	14.7	9.4

NO: Not observed.

**Table 4:** Abdominal segments I-VII (setae number, type of setae, size of the setae in μm, N = 4) of the fourth to first instar larvae of *Migonemyia migonei*.

Abdominal segments I-VII	Setae number	Type of setae	Size of the setae in the corresponding larval instar			
		Bristle	4^th^	3^rd^	2^nd^	1^st^
Dorsal intermediate	2	Barbed	35.0	12.0	5.33	2.7
Anterior ventrolateral	4	Barbed	102.5	48.0	34.65	16.0
Dorsal submedian	7	Barbed	165.0	76.0	38.70	17.4
Mid-dorsal	8	Barbed	180.0	93.3	50.65	20.0
Dorsolateral	9	Barbed	165.0	82.65	48.0	41.4
Post-ventrolateral	11	Barbed	75.0	44.0	36.0	18.7
Post-ventral	12	Barbed	30.0	13.3	10.7	NO
Ventral submedian	15	Simple	66.7	37.3	32.0	17.4
“c”-		Spine	12.0	9.35	8.00	NO

NO: Not observed.

**Table 5:** Abdominal segment VIII (setae number, type of setae, size of the setae in μm, N = 4) of the fourth to first instar larvae of *Migonemyia migonei*.

Abdominal segment VIII	Setae number	Type of setae	Size of the setae in the corresponding larval instar			
		Bristle	4^th^	3^rd^	2^nd^	1^st^
Anterior ventrolateral	4	Barbed	77.3	34.7	25.4	NO
Dorsal submedian	7	Barbed	46.7	20.0	10.7	10.7
Mid-dorsal	8	Barbed	147.15	88.0	57.3	12.0
Dorsolateral	9	Barbed	118.8	69.4	45.3	37.3
Post-ventrolateral	11	Spinulate	52.0	28.0	25.4	12.0
Post-ventral	12	Spine	22.0	12.0	6.7	8.0
Ventral submedian	15	Spinulate	57.3	26.7	25.4	9.35
“Should accessory”	a	Spine	14.7	6.7	5.4	NO
“Should accessory”	b	Spine	29.3	12.0	9.4	5.3

NO: Not observed.

**Table 6:** Abdominal segment IX (setae number, type of setae, size of the setae in μm, N = 4) of the fourth to first instar larvae of *Migonemyia migonei*.

Abdominal segment IX	Setae number	Type of setae	Size of the setae in the corresponding larval instar			
		Bristle	4^th^	3^rd^	2^nd^	1^st^
Anterior ventrolateral	4	Simple	220	152	137.5	92.0
Dorsal submedian	7	Simple	95.9	64.0	46.7	34.7
Mid-dorsal	8	Barbed	57.3	33.4	24.0	52.0
Dorsolateral	9	Barbed	57.3	36.0	24.0	16.0
Post-ventrolateral	11	Simple	97.2	48.0	38.7	21.3
Post-ventral	12	Simple	48.0	22.7	21.3	9.35
Ventral submedian	15	Simple	37.3	21.3	14.7	13.3
Internal caudal	ic	Simple	1250	950	720	560
External caudal	ec	Simple	1045	625	565	NO

NO: Not observed.

### Head

**Fig 4:**
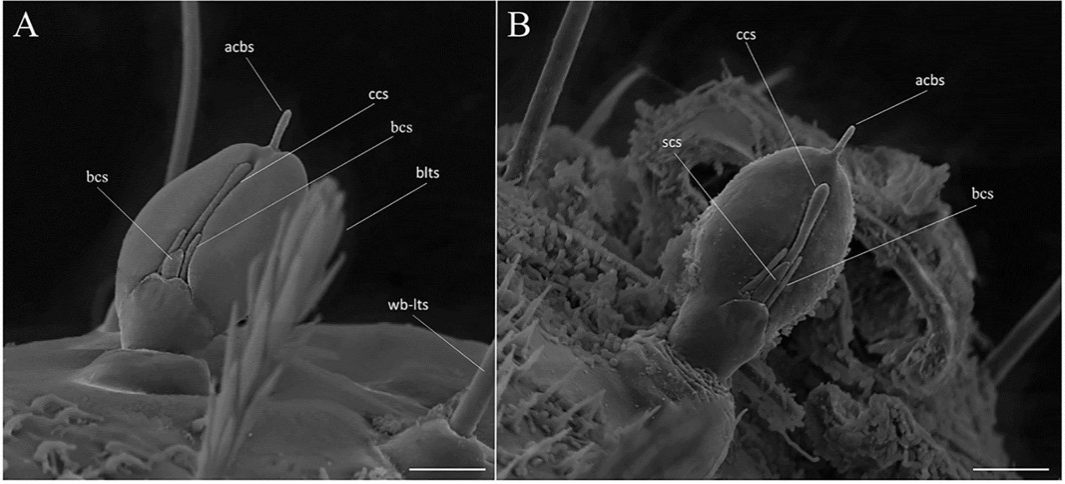
Scanning electron microscopy of the larvae of *Migonemyia migonei*. (A) antenna of the first instar larva (Scale bar: 5 μm). (B) antenna of the fourth instar larva (Scale bar: 10 μm). In A and B, a single apical clavate basiconic sensillum (acbs) is present at the apex of the antennae, and four sensilla emerge from a lateral groove: one smaller coeloconic sensillum (scs), two blunt coeloconic sensilla (bcs) above and flanking a clavate coeloconic sensillum (ccs).

The head is capsule-like, broader than it is high. The tegument is covered by thin, small spicules of the microthrichia type. On the dorsal part of the head (Fig 3A), the cephalic tagma has the following setae: (1) the anterior frontoclypeal setae (weakly brush-like trichoid sensilla subtype) with spinulate form; (2) the posterior frontoclypeal setae (brush-like trichoid sensilla subtype) with barbed shape; (3) the anterior genal setae with a simple spine form; (4) the medial genal; (5) posterior genal setae that are barbed brush-like (brush-like trichoid sensilla subtype); and, in the ventral part (Fig 3B), (6) the postgenal and (7) subgenal setae, which are simple (Table 1). All setae are inserted in small tubercles. In the first instar larva seta 1 is usually simple, but it is apparent that the projection of these setae will become barbed in subsequent instars. The antennae of *Mg*. *migonei* larvae (Fig 4A and 4B) each have a basal tubercle (socket) comprising a small and cylindrical segment fused to a second ovoid distal segment. This segment has an antennal organ equipped with three short structures in the base of the segment and a longitudinal furrow in the posterior surface that is more evident in the 1^st^ instar antennae. The central structure is wider than it is long and shorter than the lateral structures (Fig 4A and 4B). The antenna exhibits a single apical clavate basiconic sensillum at its apex and four sensilla of the *coeloconica* type emerge from a lateral groove: three smaller sensilla with blunt apexes—the middle sensillum (smaller coeloconic sensillum subtype) is wider, shorter and less cylindrical than the sensilla on either side (blunt coeloconic sensilla subtype)—and a fourth sensillum between and beneath these three which is larger and clavate (clavate coeloconic sensillum subtype—Fig 4A and 4B).

### Mouthparts

**Fig 5:**
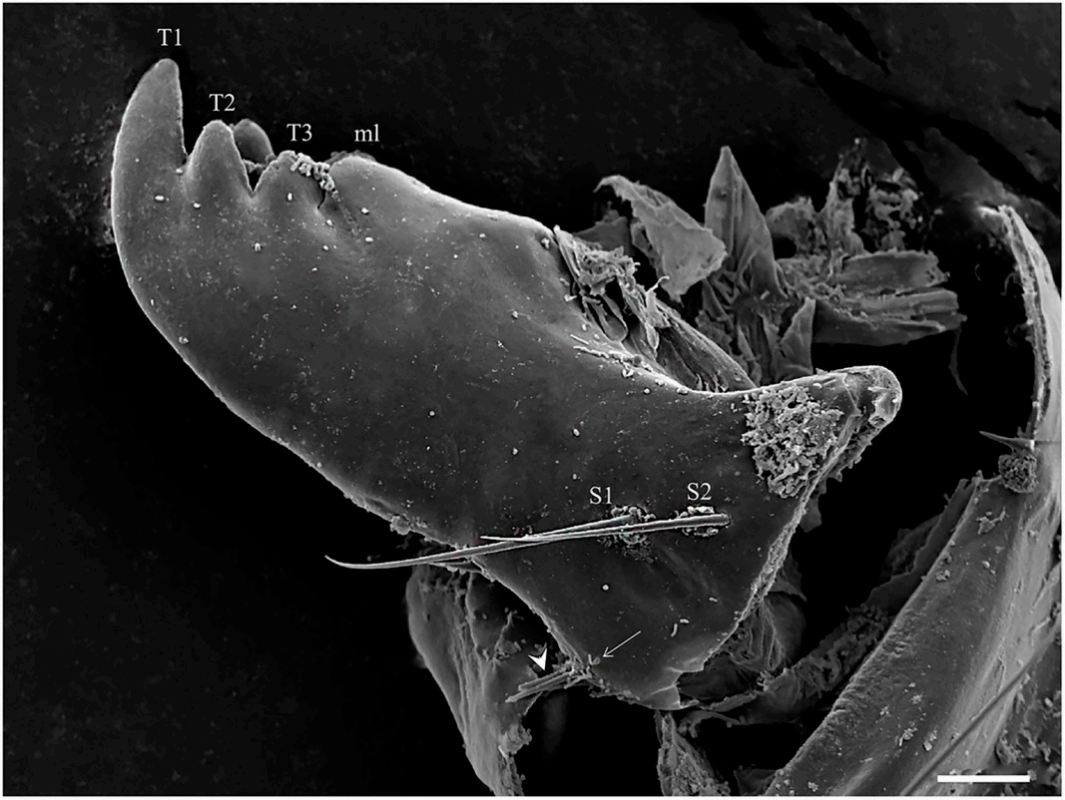
Mandible of *Migonemyia*. *migonei* larva. Apical tooth (T1), double paired median tooth (T2), proximal tooth (T3) and molar lobe (ml). Proximally, setae of the trichoid sensilla type (S1 and S2) are present; below these is a small seta (arrow); and under the margin are the tips of three other setae (arrowhead, Scale bar:10 μm).

The external part of the mouth is composed of a pair of mandibles, a pair of maxillae, labrum, and mentum. Each segmented mandible bears two simple setae in the middle of the dorsal part (S1 and S2) and a simple seta (S6) in the superior margin of the mandible similar to the setae described by Pessoa et al. [12]. In the lower part of the mandible, there are five strong teeth: a proximal tooth (T3) nearest the molar lobe (ML), an apical single tooth (T1), and double-paired median tooth (T2–which appear under light microscope as a single tooth) (Fig 5).

**Fig 6:**
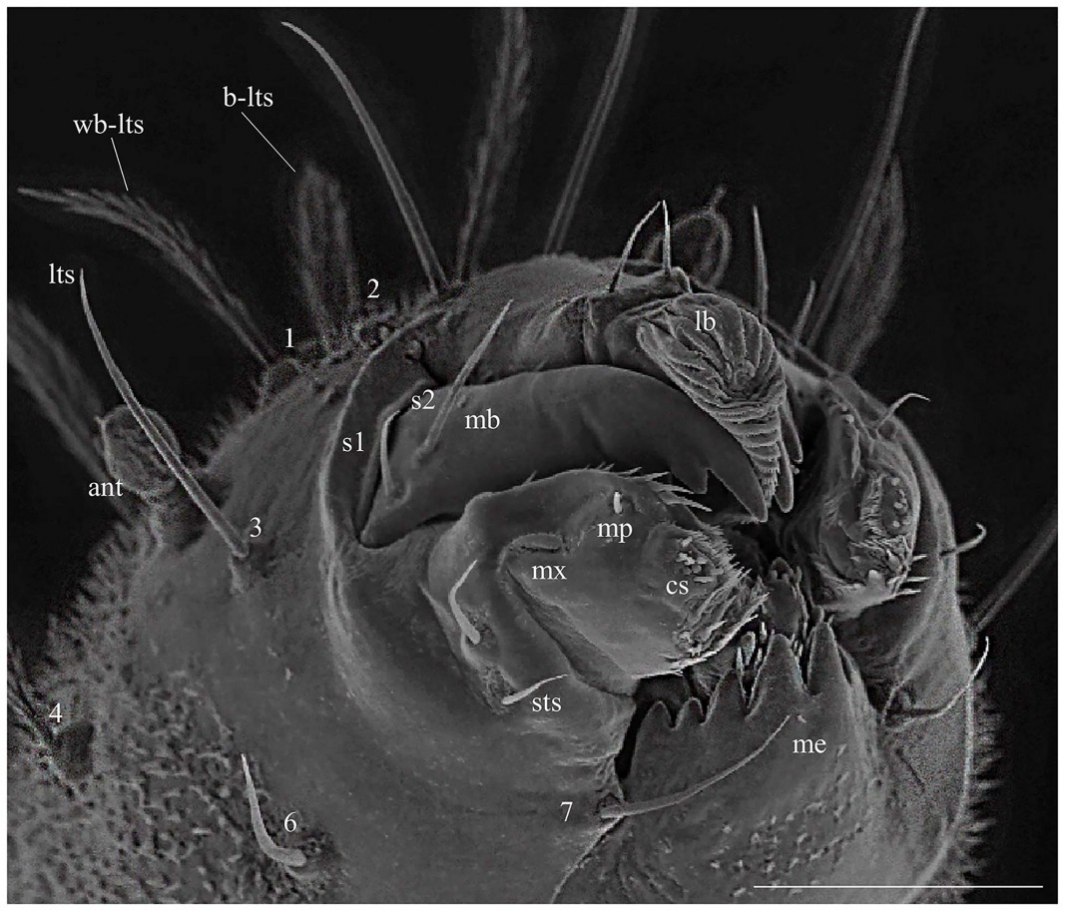
Scanning electron microscopy of the mouthparts of fourth instar larva of *Migonemyia migonei*. Head of fourth instar in ventral view. Forehead exhibits two weakly brush-like trichoid sensilla (wb-lts) inserted between and slightly forward of the antennae (ant), and long trichoid sensilla (lts) that are inserted further down toward the mouthparts, cs- campaniform sensilla; 1b –labrum; mb—mandible; mx—maxilla; me—mentum; m—mentum; s1-s6– mandible setae; mp—maxillary palpus; sts—short trichoid sensilla. The setae were numbered according to the chaetotaxy proposed herein: (1 = wb-lts) frontoclipeal anterior setae (weakly brush-like trichoid sensilla); (2 = b-lts) frontoclipeal posterior setae with barbed shape (brush-like trichoid sensilla); (3 = lts) the genal anterior seta with a simple spine form (long and bare trichoid sensilla); (4 = b-lts) the genal medial seta is barbed and brush-like trichoid; (6) the postgenal and (7) subgenal setae are simple (6 and 7 = bare trichoid sensilla; Scale bar: 20 μm).

Each maxilla has three simple setae, one (S1) in the apical dorsal part and two (S2 and S3) in the proximal part (Fig 6). There is a maxillary process in the middle of this structure. In the margin of the dorsal part, there is an array of small and sparse combs of spines, similar to those found in the maxilla of *Ev*. *lenti* and *Ev*. *carmelinoi* [12]. At the apex, there are papilliform and trichodea sensillae (spinous hairs). On the upper side, there is a row of small setae. The ventral surface of the labrum is covered with parallel, transverse rows of finger-like combs of setae; the dorsal side has two pairs of very small simple setae. The clypeus has two pairs of simple setae, the distal pair is small and the apical pair is large (Fig 6).

**Fig 7:**
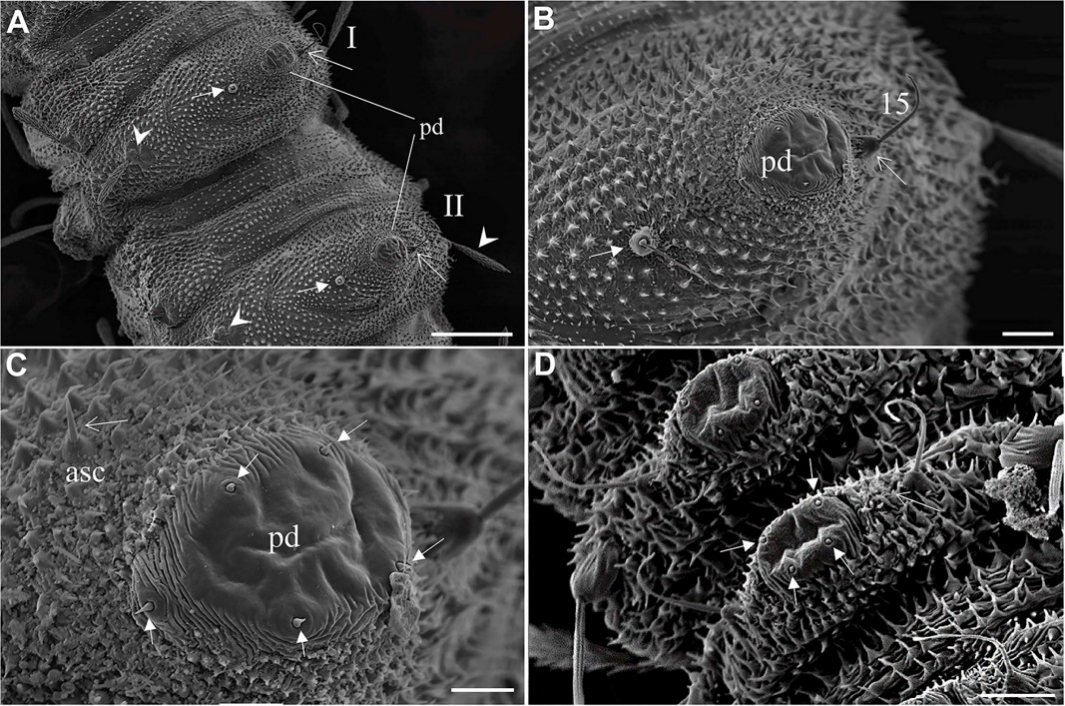
Scanning electron microscopy of the ventral part of abdomen and pseudopoda of *Migonemyia migonei*. (A–C) mature larvae in ventral view (Scale bars: 100 μm, 20 μm and 10 μm respectively). (D) first instar larvae in ventral view: asc—accessory setae; pd—pseudopod; * ampliation accessory setae; brush-like trichoid sensilla (arrowhead); long and bare trichoid sensilla (thin arrow); 15 –ventral submedian. (Scale bars: 10 μm).

**Fig 8:**
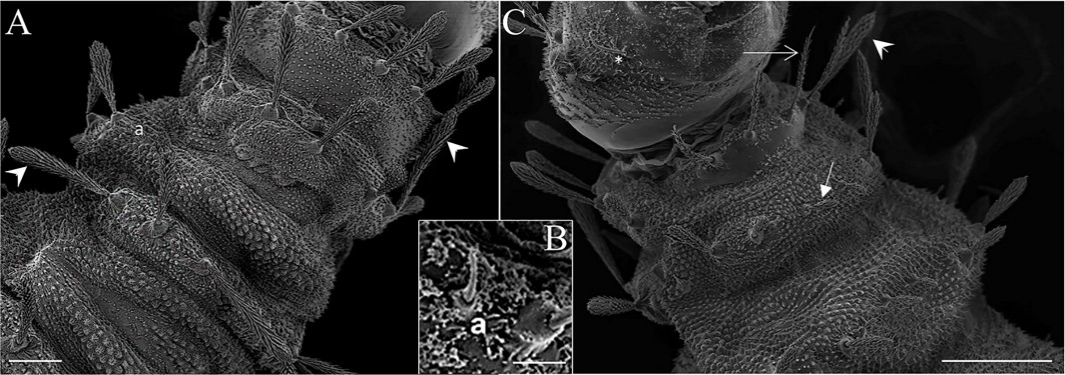
Scanning electron microscopy of the mature larva of *Migonemyia migonei*. (A) prothorax and mesothorax of the fourth instar larva in dorsal view (Scale bars: 50 μm). (B) a—accessory setae. (C) head, prothorax and mesothorax of the fourth instar larva in ventral view, brush-like trichoid sensilla (arrowhead); weakly brush-like trichoid sensilla (thin arrow); * long and bare trichoid sensilla (Scale bars: 100 μm).

**Fig 9:**
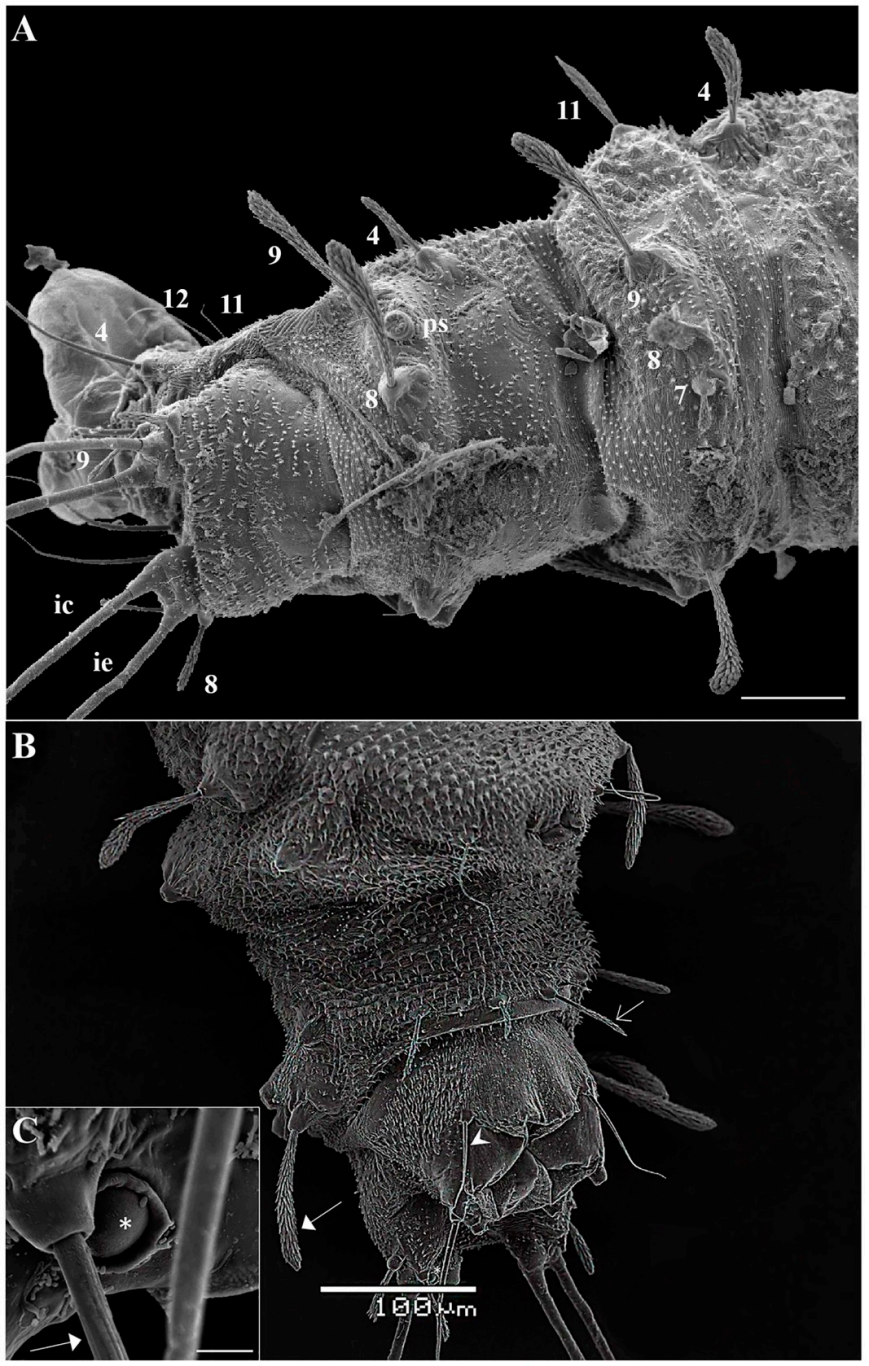
Scanning electron microscopy of the fourth instar larva of *Migonemyia migonei*. (A) abdominal segments seven to nine of the fourth instar in dorsal view (Scale bars: 50 μm), and (B) ventral view (Scale bars: 100 μm). (C) increased area, ventral view (Scale bars: 5 μm). Setae numbered according to the chaetotaxy proposed in this study ec- external caudal; ic—internal caudal; ps—posterior spiracle; *campaniform sensilla; anterior ventrolateral (4); submedian dorsal (7); mid—dorsal (8); dorsolateral (9); post-ventrolateral (11); post-ventral (12); (Scale bars: 50 μm and 100 μm, respectively); brush-like trichoid sensilla (thick arrow); weakly brush-like trichoid sensilla (thin arrow); long and bare trichoid sensilla (arrowhead).

The thorax has three segments, the prothorax has the appearance of two segments, and the setae of the meso and metathorax are homologous with the posterior setae of the prothorax. Chaetotaxy follows the same pattern of setae as classified by Ward [27] and is presented in Tables 2 and 3, and Figs 7 and 8. The anterior spiracles are conical and have eight to nine papillae in the fourth instar larva (Fig 9A), five to six in the third instar larva, and four in the second instar larva (Fig 9B). The images that we obtained were not clear enough to compare or count anterior spiracles of the first instar larva.

### Chaetotaxy of prothorax

The tergite has two rows of setae. The first row has three pairs of setae: the dorsal internal, dorsal intermediate and dorsal external. The second row has two setae: the dorsal submedian and the mid-dorsal. The pleura has two setae—the anterior ventrolateral and the dorsolateral—which appear to change position in the larvae; these setae are similar, barbed or brushed-like, and differ only slightly in size (Table 2). A hyaline spine-seta is present between the first and second row of setae, usually near the ventrolateral setae. The sternite also has two rows of setae. The first row has two similar pairs of setae: the ventral external and ventral internal—these are a little less barbed than the dorsal setae. The second row has seven pairs of setae that differ in size and shape: the basal, post-ventrolateral, post-ventral, mid ventral, ventral intermediate, ventral submedian, and b setae (Table 2). The meso and metathorax lack the first row of setae present in the prothorax tergite and sternite, but they do exhibit a row of setae with the same features as the second row of setae in the prothorax (Table 3).

The setae of the abdomen exhibit the distribution proposed by Ward [27], but with seta 10 absent in each segment. Segments one to seven are homologous, and of similar size and shape. In the anterior part of the pseudopodium, there is a simple pair of setae, which are similar in size to setae 11 and 12, but other authors do not ascribe taxonomic value to these setae; we have named this pair of setae “c” (Table 4 and Fig 8C). The eighth and ninth segments are darker. The posterior spiracles are conical, with ten papillae. The shape and measurements of the setae are detailed in Fig 8A and 8B and Tables 5 and 6. Chaetotaxy of abdominal segments one to seven is as follows: In the tergites, the pairs of setae are not grouped; the anterior dorsal intermediate setae are much smaller than the others, the anterior ventrolateral setae are in the border of the pleura, and there is a row of paired setae on the dorsal submedian, mid-dorsal, and dorsolateral parts of the tergite, and in the border of the pleura. All of these setae are barbed and differ in size (Tables 4–6). The sternites (Fig 8) have large pseudopodia with a few simple setae, the post-ventrolateral and the post-ventral setae are both very small and simple and there is a large and simple ventral submedian seta. In the anterior part of the pseudopodia, there is a pair of setae which are very similar to the post-ventrolateral and the post-ventral setae that we have named the "c" setae. Abdominal segments eight and nine lack pseudopodia. Abdominal segment nine ends in two tubercles, each of which bear a caudal filament (Fig 8A). A large campaniform sensilla is present in the ventral side of these tubercles (Fig 9B). The posterior spiracle has 11–14 papillae.

### Other larval instars

**Fig 10:**
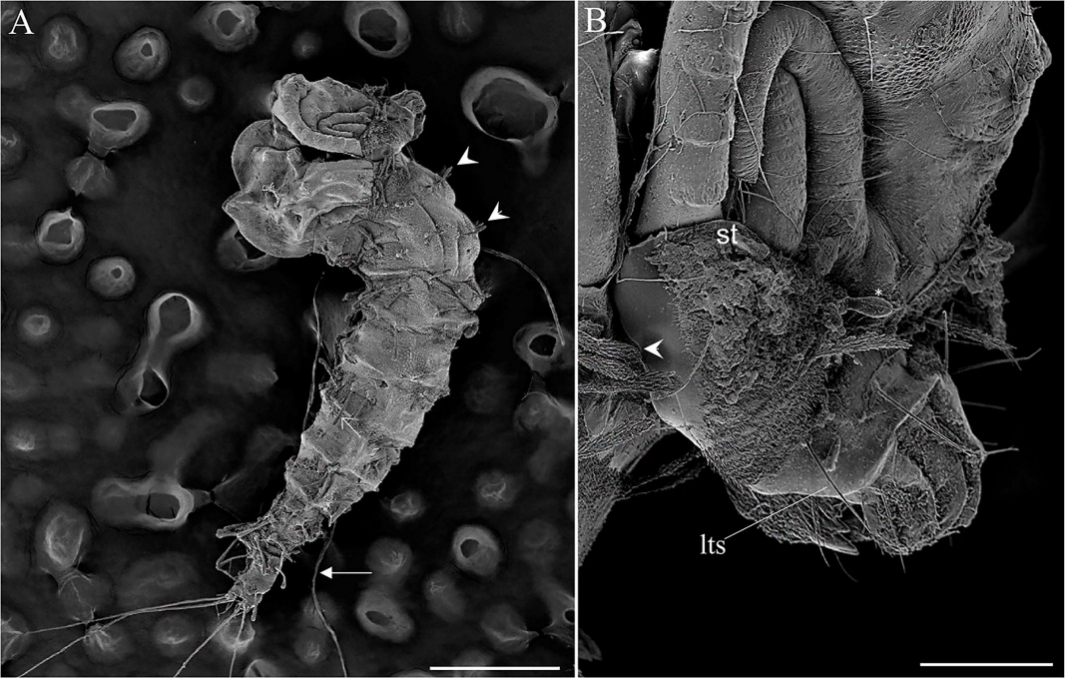
Scanning electron microscopy pupae of *Migonemyia migonei*. (A–B) emerging from a mature larva lateral view; suture (st) opened from larva tagma head; * antennae; caudal filament (arrow); long and bare trichoid sensilla (lts); brush-like trichoid sensilla (arrowhead); (Scale bars of A and B: 500 and 100 μm, respectively).

The body sizes (length from the head to end of the ninth abdominal segment and maximum width at the metathorax) of the third to the first instar larvae are, respectively: 1.7 and 0.27; 0.94 and 0.13; 0.56 and 0.09 mm. The first instar is easily identified by the presence of a unique pair of caudal filament (long, multiporous trichoid sensilla—Fig 2B) and the absence of some bristles in the prothorax (seta 3, a, b and 10; Table 2) and some bristles in the pro, meso and metathorax (b and 10; Table 3), and the presence of the egg buster in the head (Fig 3D). Setae 6 and 14 of the prothorax are simple in the first instar and barbed in the other instars. Setae 11 and 15 of abdominal segment eight are simple but become almost barbed in the other instars. Chaetotaxy for the other instars differs with respect to size but is otherwise the same as the chaetotaxy for the fourth instar (Tables 1–6). The pupa emerges from a Y shaped suture in the head of the mature larva (Fig 10A and 10B).

### Pupa description

**Fig 11:**
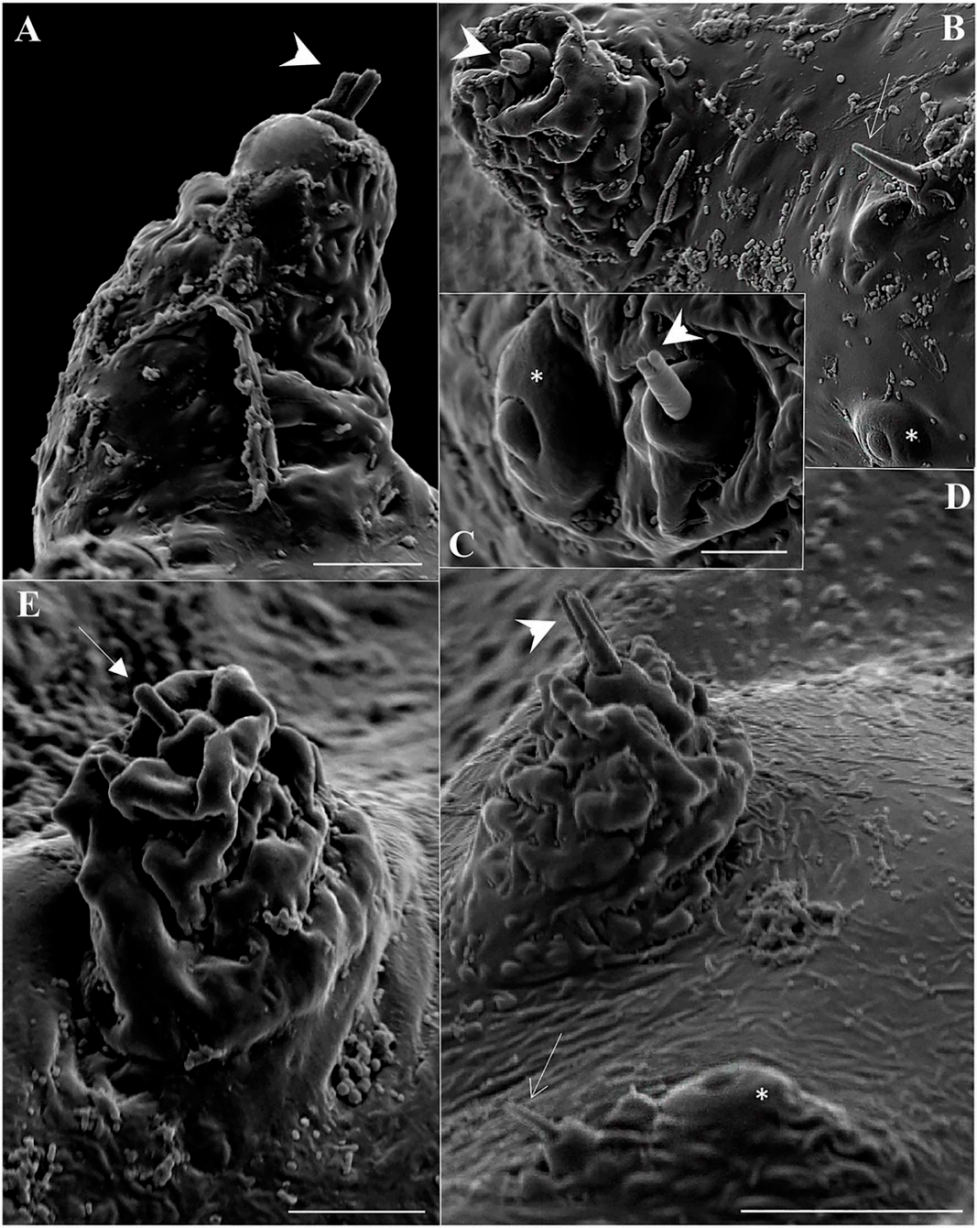
Scanning electron microscopy of the pupa of *Migonemyia migonei*. (A) Metathorax setae 1T in dorsal view (Scale bar: 10 μm). (B) Prothorax setae 1P and 2P in dorsal view (Scale bar: 20 μm). (C) Metathorax setae 3T in dorsal view (Scale bar: 5 μm). (D) Mesothorax setae 1M and 2M in dorsal view (Scale bar: 20 μm). (E) Metathorax setae 2T in dorsal view (Scale bar: 10μm). Setae numbered according to the chaetotaxy proposed in this study: 1P, 1T, 1M and 3T; forked-apex short trichoid sensilla (arrowhead); 2P and 2M; blunt short trichoid sensillum (thick arrow); short trichoid sensillum (thin arrow)* campaniform sensilla.

**Fig 12:**
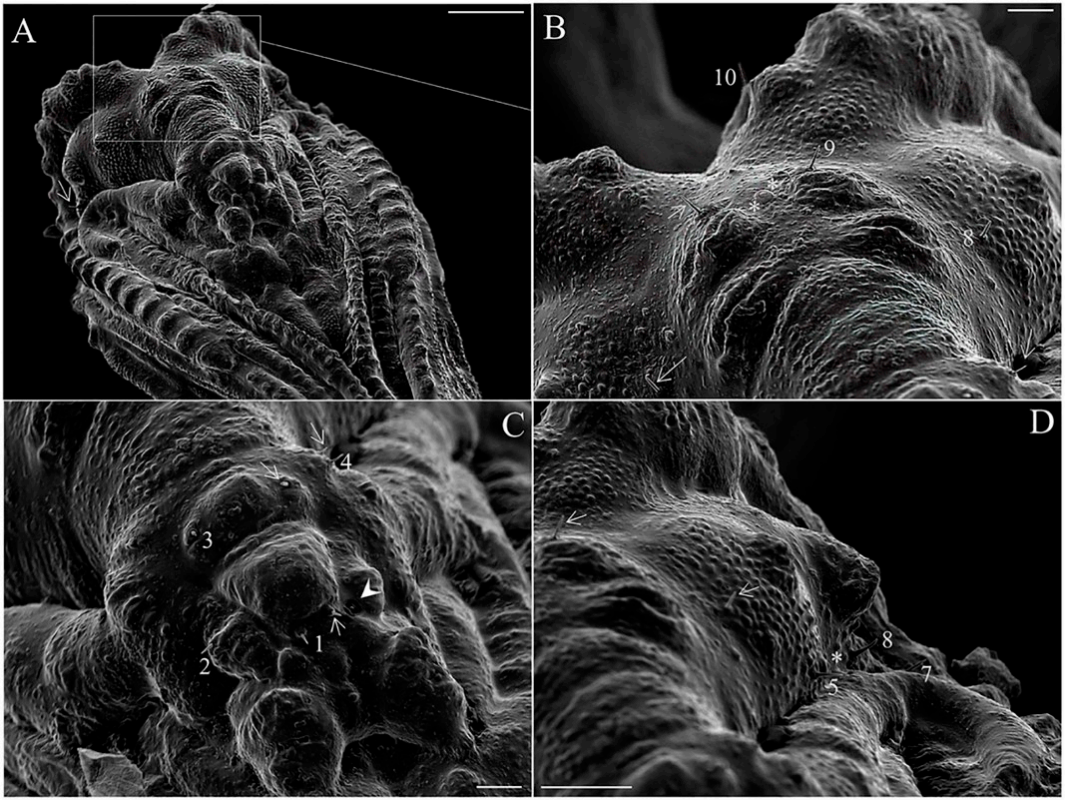
Scanning electron microscopy of the pupa of *Migonemyia migonei*. (A) head of the pupa in ventral view (Scale bar: 100 μm). (B–D) head of the pupa in ventral view (Scale bars: 20 μm). Setae numbered according to the chaetotaxy proposed in this study: Clypeal inferior (1); Palpal seta (2); Superior clypeal (3); Inferior frontal (4); Medial postocular (5); Internal postocular (6); External postocular (7); Medial frontal (8); Superior frontal (9); Vertical (10); short trichoid sensillum (thin arrow); * campaniform sensilla; sensilla coeloconicum (arrowhead);

**Fig 13:**
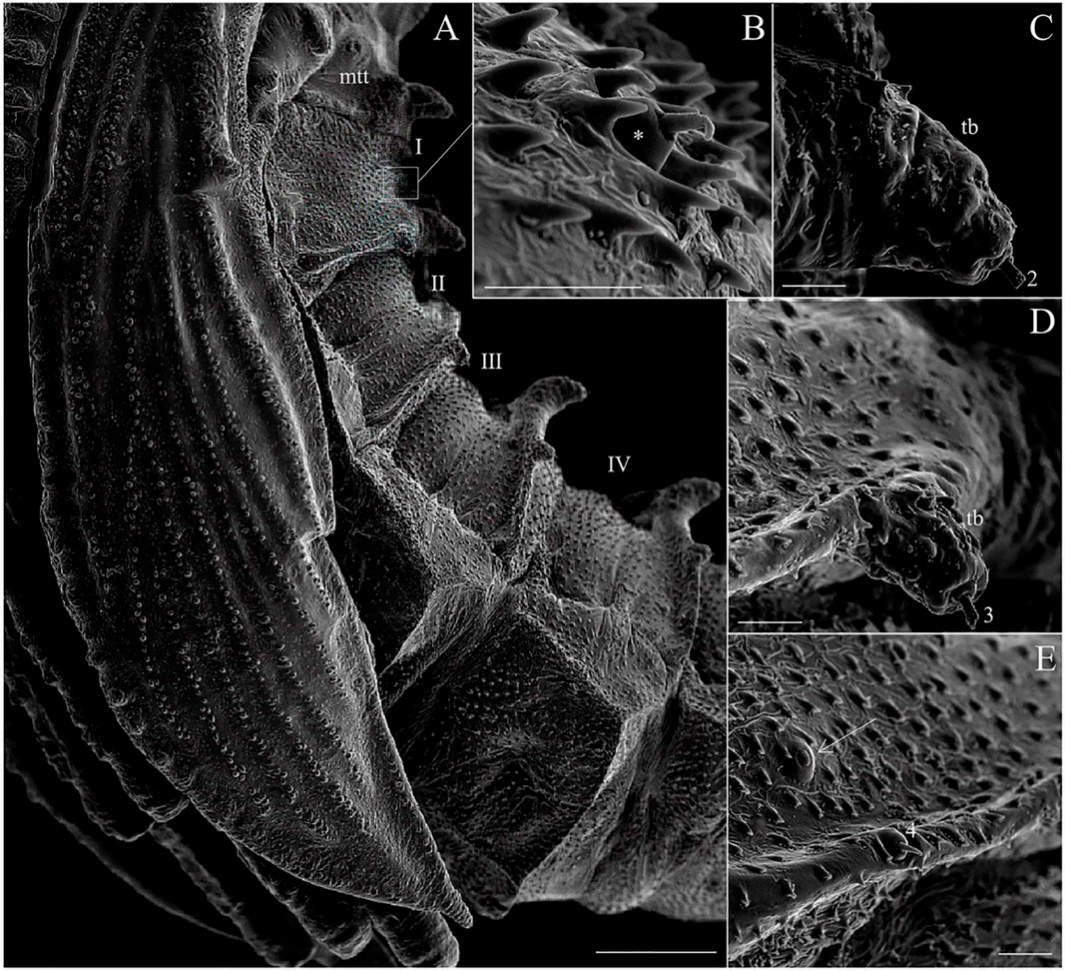
Scanning electron micrograph of the pupa of *Migonemyia migonei* in lateral view. (Scale bars: A: 100 μm; B–D: 10 μm). as—abdominal segment; swv—sheath of wing venation; tb—tubercle; arrow pointing to campaniform sensilla; * area of increased magnification showing seta dorsal anterior (1); Internal posterior dorsal (2); External posterior dorsal (3); Laterodorsal (4).

**Fig 14:**
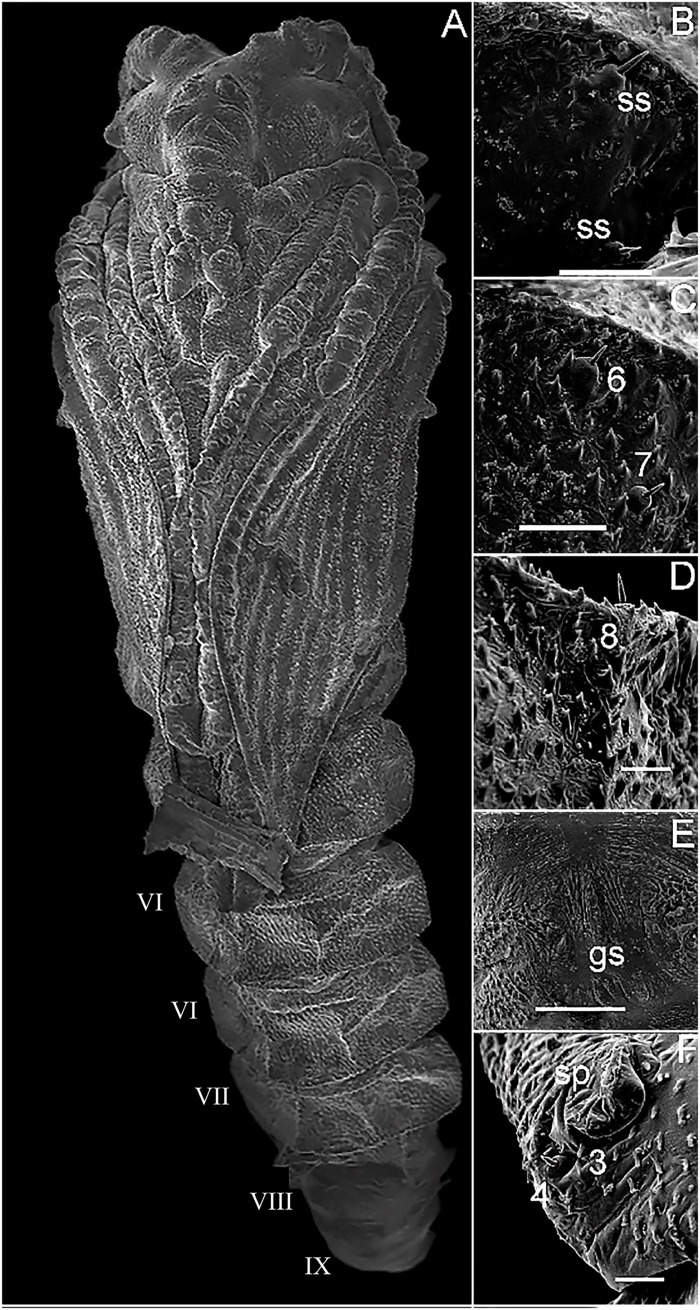
Scanning electron microscopy of the pupa of *Migonemyia migonei*. (A) pupa in ventral view; (B, C and D) ventral abdominal segment of setae. (E) genital opening sheath. (F) part of abdominal segment VIII with posterior spiracle. as—abdominal segment; gs—genital sheath; sp—spiracle. External posterior dorsal (3); Laterodorsal (4); External posterior ventral (6); External anterior ventral (7); Internal posterior ventral (8).

**Fig 15:**
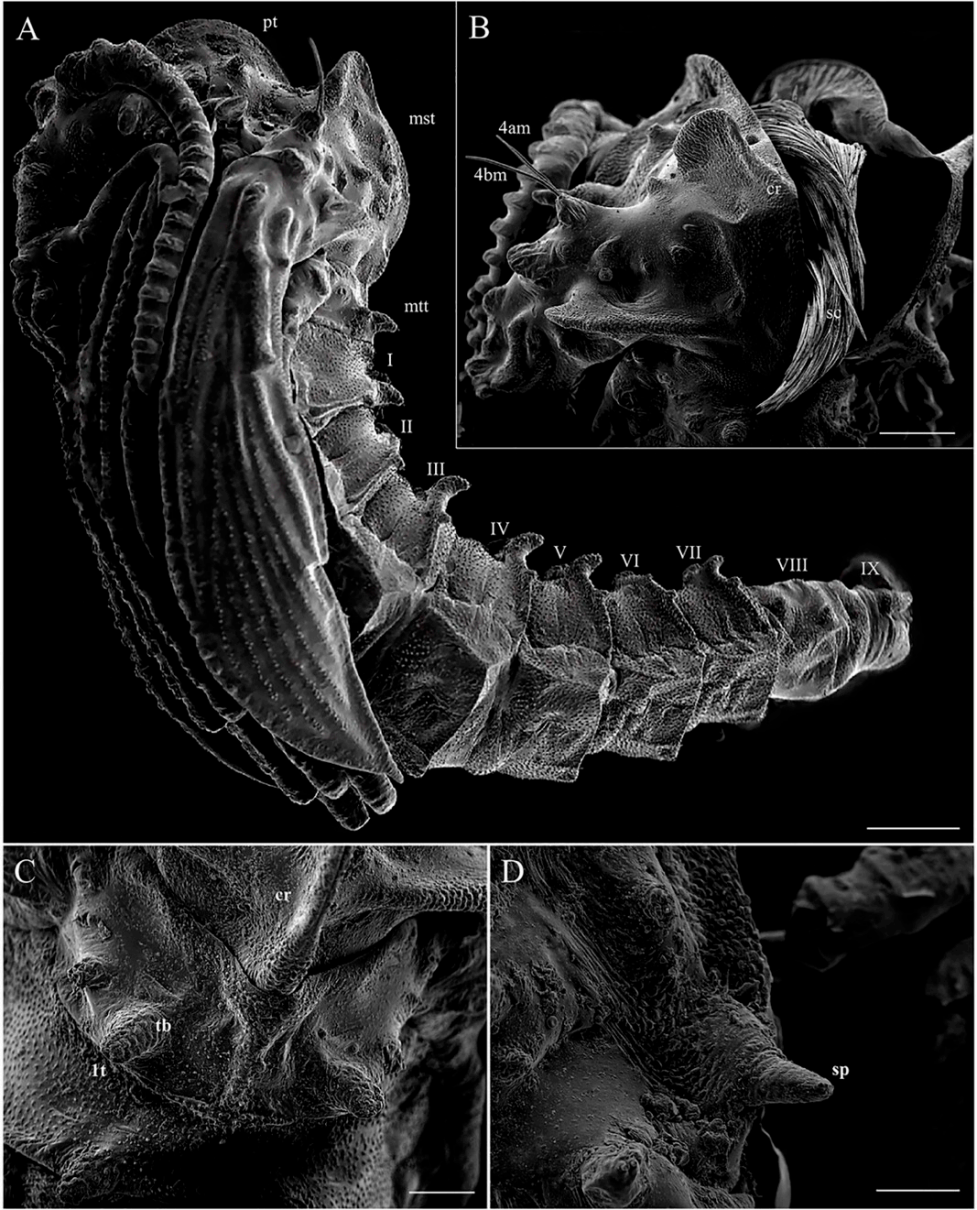
Scanning electron microscopy of the pupa of *Migonemyia migonei*. (A) pupa in lateral view (Scale bar: 200 μm). (B) opening of pupa crest (Scale bar: 100 μm). (C) metathorax. (D) superior spiracle (C and D scale bars: 50 μm). Abbreviations: cr—crest; mst—mesothorax; mtt—metathorax; pt—prothorax; sc—scales (hairs) of the pharate adult; sp—spiracle; tb—tubercle.

**Fig 16:**
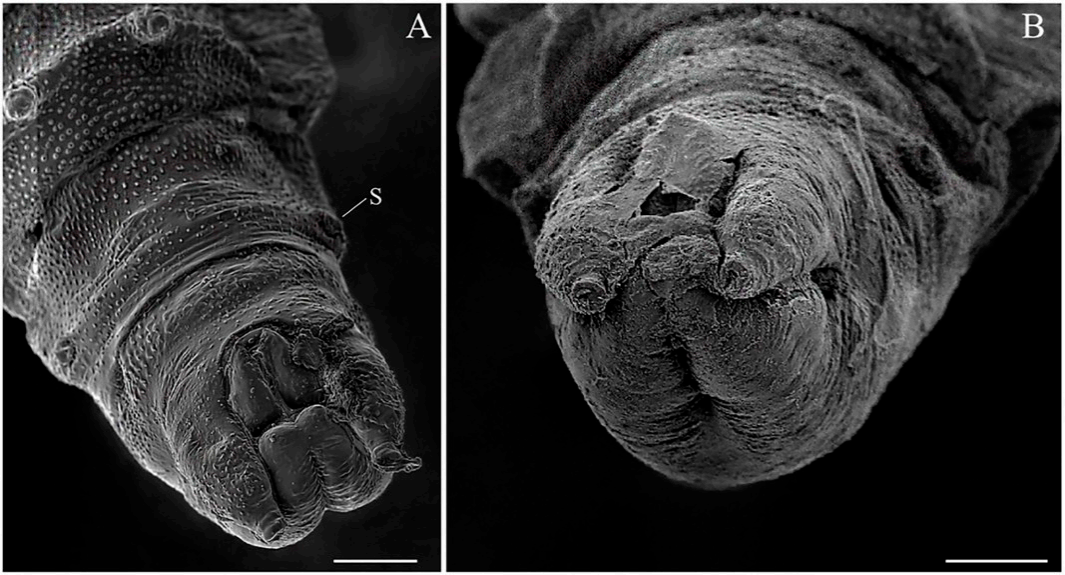
Scanning electron microscopy of the pupa of *Migonimyia migonei*. (A) Female pupa in ventral view. (B) Male pupa in ventral view (Scale bars of A and B: 50 μm). s—spiracle.

The pupa of *Mg*. *migonei* is claviform and divided by the cephalothorax and abdomen (Figs 11A, 12, 13 and 14). The pupa tegument has some small ornaments, such as small spines and setae (described in Table 7), and the body is covered by several small rounded tubercles (Figs 11–13 and 15). The pharate female pupa is longer (2.24 mm, n = 5) than the pharate male pupa (2.05 mm, n = 5) (Fig 16).

**Table 7:** Head chaetotaxy for pupa de *Migonemyia migonei*.

	Setae number	Type of setae	Terminology
**Head**	1C	Basiconic	Clypeal inferior
	2C	Basiconic	Palpal seta
	3C	Basiconic	Clypeal superior
	4C	Basiconic	Frontal inferior
	9C	Basiconic	Frontal superior
	10C	Basiconic	Vertical
	8C	Basiconic	Frontal medial
	5C	Basiconic	Postocular medial
	6C	Basiconic	Postocular internal
	7C	Basiconic	Postocular external

The cephalic sheath with antennal impressions shows outlines of all flagellomeres of the pre-imaginal stage. Mouth part sheath is smooth; clypeal sheath is conspicuous; sexual dimorphism is present in the maxillary sheath which is shorter than the labrum-epipharynx and hypopharynx sheaths in males; head chaetotaxy with very small spines (Fig 12) as numbered in Table 7. Thorax with a large, Y-shaped longitudinal crest in the middle of the dorsal side. Pro and mesothorax have a pair of prominent tubercles, and the metathorax has two larger pairs (Table 8, Fig 11). There is a pair of spiracles (ventilatory orifice, per [8]) in the prothorax. The prothorax has 2 + 2 small setae in each spiracle and a campaniform sensilla. The mesothorax has 3 + 3 pairs of setae, one pair are long, chaotic, prealar setae with blunt tips, (length 0.15 ± 0.008 mm, n = 5), the others are stout and originate from tubercles; the mesothorax also has a large mesonotal tubercle with a continuous border (Fig 11B); the mesotonal tubercle is considered herein to be part of the Y arm of the longitudinal crest, which is scattered with small, rounded tubercles. The metathorax has four pairs of setae, some associated with tubercles, and there is a small bifurcation in the tip of setae 1T and 3T (Table 8, Fig 11D and 11F). The ventral side of the thorax has leg and wing sheaths and marked wing venation, with a row of rounded tubercles in each stamped venation (Figs 11A, 13A and 14A).

**Table 8:** Thorax chaetotaxy for pupa de *Migonemyia migonei*.

Thorax	Setae number	Type of setae	Terminology
Prothorax	1P	Basiconic	Protoracic superior
	2P	Basiconic	Prothoracic medial
	3P	Absent	Absent
Mesothorax	1M	Styloconic	Mesothoracic inferior
	2M	Styloconic	Mesothoracic medial
	3M	Absent	Absent
	4A - B M	Chaetic	Pre-alar
Metathorax	1T	Styloconic	Metathoracic internal
	2T	Styloconic	Metathoracic medial
	3T	Basiconic	Metathoracic external
	4A - B T	Basiconic	Pre-halter

**Fig 17:**
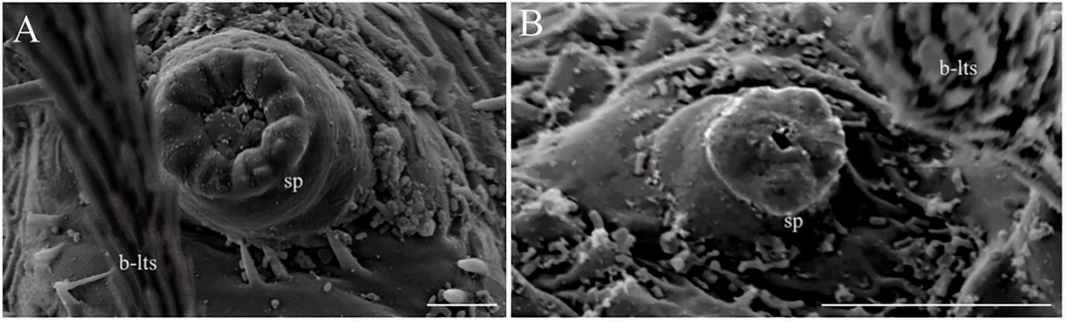
Scanning electron microscopy of the larvae of *Migonemyia*. *migonei*. (A) anterior spiracles of the third instar larva (Scale bar: 5 μm); (B) anterior spiracles (sp) of the second instar larva brush-like trichoid sensilla (b-lts, Scale bar: 10 μm).

The tegument of the abdomen is covered by several small, spiniform tubercles. There are nine segments, and the width of each segment is nearly twice its length. Segments diminish gradually in size towards the distal region (Fig 17A) and become discrete lateral projections in the pleural sheath. The last segments show sexual dimorphism, with a posterior spiracle in the eighth segment. Segments I–VII have two pairs of median dorsal tubercles. Abdominal segments I–II have a tergum with four pairs of setae, and pleura and sternum are covered with the thoracic appendage sheaths. Abdominal segment III has a tergum and sterna with four pairs of setae on each side that are similar in shape and location to the setae on abdominal segments IV–VII; pleura and sterna III are partially covered by the thoracic appendage sheaths (Table 9). In abdominal segments IV–VII, each tergum has four pairs of setae distributed in a similar fashion to the setae of the previous segments. Each sternum has four pairs of setae. In abdominal segment VIII, males and females both have two pairs of setae on the terga, two pairs of setae on the sternum and two pairs on the spiracle (Fig 14F); these are all very small basiconic setae. Abdominal segment IX is covered by the larval exuviae (as is VIII), but when uncovered, sexual dimorphisms were found. Male pupae possess two lobes on each side—one simple, covering the lateral lobe, and the other marked divided, with the gonostylus and gonocoxite. In females, there are two simple and short lobes on each side—one covering the oviscape and the other covering the cercus, though without setae (Fig 16), and the opening of the genital sheath is discreet (Fig 14E). Abdominal chaetotaxy can be seen in Table 9, Figs 13 and 14.

**Table 9:** Abdomen segments chaetotaxy for pupa de *Migonemyia migonei*.

Abdomen segments	Setae number	Type of setae	Terminology
I-VII	1	Basiconic	Dorsal anterior
	2	Styloconic	Dorsal posterior internal
	3	Styloconic	Dorsal posterior external
	4	Basiconic	Laterodorsal
	8	Basiconic	Ventral posterior internal
	7	Basiconic	Ventral anterior external
	6	Basiconic	Ventral posterior external
	9	Basiconic	Ventral anterior internal
VIII	1	Basiconic	Dorsal superior
	2	Basiconic	Dorsal inferior
	3	Basiconic	Lateral
	4	Basiconic	Lateral
	5	Basiconic	
	6	Basiconic	

## Discussion

The exochorion pattern of *Mg*. *migonei* is polygonal and was first described by Barreto [29] using only light microscopy, and subsequently redescribed by Fausto et al. [23] who used SEM. This polygonal pattern of exochorion ornamentation is found in 28 other Neotropical sand fly species [14, 30], distributed in nine genera of different subtribes. This characteristic is probably phylogenetically significant, but according to Perez & Ogosuku [31] the exochorion pattern does not reflect phylogenetic relationships based on adult characteristics. Ward & Ready [32] and Costa et al. [33] suggest that sand fly exochorion patterns may differ according to breeding site environment. Using SEM, Bahia et al. [7] observed that the eggs of *Ny*. *intermedia* and *Ny*. *whitmani* present an exochorion pattern this is different than the eggs of *Mg*. *migonei*. Instead of presenting ornaments with polygonal reticulation of alternating transversal rows of generally rectangular parallel cells or square to polygonal cells, the eggs of *Ny*. *intermedia* and *Ny*. *whitmani* present ornaments consisting of parallel ridges that cover the entire exochorion. This exochorion pattern was previously observed in these same *Nyssomyia* species by Barretto [29], who used light microscopy and described the pattern as “connected ridges”. Subsequently, Pessoa et al. [12] described the exochorion of *Ev*. *Carmelinoi*. The *Evandromyia* genus is phylogenetically close to *Migonemyia*, but knowledge about sand fly zootaxonomy nevertheless remains scarce.

The chaetotaxy and morphological structures of Neotropical phlebotomine larvae have been discussed previously by several authors [4, 7, 8, 12, 14–16, 27, 29, 32, 34–38]. *Mg*. *migonei* larvae observed under SEM have characteristics similar to the larvae of *Ny*. *intermedia* and *Ny*. *whitmani* [7]. These characteristics include: the general aspect of the head, the somewhat prognathous position of the mouthparts, the peculiar “volcanic cone” shape of the egg buster [7], the cylindric body shape, and the types and position of bristles. These characteristics have also been observed in some species from the Lutzomyiina and Sergentomyiina subtribes, such as *Lutzomyia longipalpis*, *Lu*. *cruciata*, *Ev*. *carmelinoi* [12], and *Micropygomyia chiapanensis* [4].

Trichoid sensilla of various subtypes are the most common type of sensilla found on larval and adult phlebotomine sand flies and other Diptera [7, 39, 40]. *Mg*. *migonei* larvae have short and long trichoid sensilla surrounding the base of the anal lobes and on the lateral sites of the prolegs, and these are similar to trichoid sensilla previously observed at the same sites in the larvae of *Ny*. *intermedia* and *Ny*. *whitmani* [7]. The larvae of these *Nyssomyia* species also exhibit similarities in typology and pattern of sensillary distribution, such as: brush-like trichoid sensilla located in front of the egg buster and on the lateral and dorsal aspects of the body segments, trichoid sensilla on the apex of the head, short trichoid sensilla on the mouthparts, weakly brush-like trichoid sensilla inserted between and slightly forward of the antennae, and long trichoid sensilla inserted further down towards the mouthparts.

In addition to these sensillary types, other sensilla present in *Mg*. *migonei* larvae are also evident in *Ny*. *intermedia* and *Ny*. *whitmani* larvae; such as, one apical clavate basiconic sensillum on the apex of the antennae, and one clavate coeloconic sensilla and three short blunt coeloconic sensilla implanted on the proximal region of the antennae [7]. In *Ny*. *intermedia* larvae, the latter sensillary subtype was examined under higher magnification by Bahia et al. [7] and observed to have wall pores (multiporous clavate coeloconic sensilla; SW-sensillum subtype). At the same site in *Lu*. *longipalpis* larvae, Pessoa et al. [11] identified a similar sensory subtype with wall pores, previously designated as “multiporous papilla”. Sensilla similar to the apical clavate basiconic sensillum and the clavate coeloconic sensilla were also observed by Oca-Aguilar et al. [15] in the larval antennae of the *Lu*. *cruciata* sand fly.

The antennal pattern of mature *Mg*. *migonei* larvae under SEM was described by Pessoa et al. [11], and the earlier stages described here match category *iv* of the Leite & Williams [5] proposal for antennae shape. In the mouthparts, the teeth of the mandibles present distinctive characteristics that usually go unnoted because the mandible is ordinarily described in lateral view. The chaetotaxy of the *Mg*. *migonei* thorax is quite similar to that of *Ev*. *carmelinoi* and *Ev*. *lenti*, a genus that is close to *Mygonemyia* and has been described using a similar methodology. In the thorax, only the shoulder accessory b seta is evidently different, being very small, bifid or trifid, semi-barbed and twice as large in *Mg*. *migonei* relative to *Ev*. *carmelinoi*, and absent in *Ev*. *lenti*.

The anterior spiracles of the *Mg*. *migonei* population obtained from Ceará State in Brazil possess a few more papillae (eight to nine) than specimens from Mérida State in Venezuela, which have seven [9]; *Ev*. *carmelinoi* and *Ev*. *lenti* also have eight anterior spiracles [12]. The number of papillae of the posterior spiracle is similar for both *Mg*. *migonei* populations as well as for *Ev*. *carmelinoi* and *Ev*. *lenti*. Dorsal submedian setae 7 and 8 of the meso and metathorax and abdominal segments I-VII of *Mg*. *migonei* are similar in size to those of *Ev*. *carmelinoi* [12], and, in both cases, these setae are twice as large as the same setae in *Ev*. *lenti* [12]. Setae 11 and 12 of *Mg*. *migonei* are barbed and twice as large as the same simple setae in *Ev*. *carmelinoi* and *Ev*. *lenti* [12]. The last segment also has slight differences: setae t and 11 in *Mg*. *migonei* are bigger than in *Ev*. *Lenti*, and seta 12 in *Mg*. *migonei* is only half the size of its counterpart in *Ev*. *lenti* [12]. A large campaniform sensilla present in the ventral side of each tubercle of implantation of the caudal setae has not been previously described and is duly registered here.

The caudal filaments present in the last larval segment of *Mg*. *migonei* and other sand flies are a subtype of long trichoid sensilla that has a multiporous wall (SW-sensilla), and is therefore classified as an olfactory sensory structure [41, 42]. Olfactory sensilla exhibit very particular characters in their superficial microstructure—multiporous walls (SW-sensilla) or walls with longitudinal grooves (DW-sensilla) [42–44] and these characteristics determine the generic olfactory function. The way in which sensilla respond to odor molecules can only be determined using electrophysiological bioassays, such as Single Sensillum Recording coupled to Gas Chromatography (SSR-GC), which is an efficient method for isolating potential insect attractants and allows the action potentials of odor receptor neurons (ORNs) present in each type of olfactory sensilla to be recorded *in situ* [45]. This highlights the great importance of previous studies that have used scanning electron microscopy to map the sensillary topography and identify the olfactory sensilla and their precise location. SSR-GC bioassays facilitate the accurate orientation and targeting of electrodes and odor pulses, especially in the analysis of insects with very small, pilose antennas, like those of sand flies and other Diptera.

Different sand fly species have different sculpture of their pores in their caudal filaments. For example, *Ny*. *whitmani* larvae have deep caudal filament pores distributed within wall grooves that are set well apart, whereas *Ny*. *intermedia* have superficial pores that are well separated and are not found in well-defined grooves [7]. *Ev*. *lenti* larvae have fewer pores in their caudal filaments, and these are distributed along thin, longitudinal ridges that run in parallel and are set close together [11]. *Mg*. *migonei* larvae have caudal filaments with a larger number of deep pores distributed between non-parallel ridges that interconnect. Differences in the distribution patterns of pores in caudal filament walls may serve as important characters in future taxonomic and phylogenetic studies of larvae, and thereby improve our understanding of the evolution and allopatric speciation process in Phlebotominae.

This study presents the first images of the pupa emerging from the Y suture of the head, and the subsequent rupture in the middle of the dorsal part of thorax that results from the contraction and inflation of the pupa. Only a few pupae from Neotropical sand fly species have been described in detail using 3D images from SEM; however, all the head setae, small spines, and basiconics are homologous among described Neotropical pupae [8, 13–16]. Nevertheless, some minor differences are evident in the thorax: Setae 1P and 2P are basiconic, 1P is bifid and stout, and implanted in a tubercle. In some species, setae 1P and 2P are styloconic, or at least one of them is, as is the case for *Da*. *beltrani* [8], *Mg*. *chiapanensi* [14], *Lu*. *cruciata* [15], *Ny*. *umbratilis* [16], although this characteristic is not phylogenically significant for Lutzomyiina. The absence of the 3P seta in *Mg*. *migonei* is probably an apomorpy in this species, at least with respect to the other species discussed in this paper. Abdominal segments I–VII have two pairs of median dorsal tubercles, one pair being large and conspicuously curved backwards. *My*. *chiapanensis* [14] and *Lu*. *cruciata* [15] possess these tubercles in discrete form, but these tubercles are not present in *Da*. *beltrani* [8] or *Ny*. *umbratilis* [16]. Setae 5 are absent in *Mg*. *migonei* but present in other described pupae, with the exception of *Ny*. *umbratilis* [16].

In the present study, the immature stages of *Mg*. *migonei* possess some discrete or evident structures that can be used as apomorphies to elucidate phylogenetical relationships and thereby improve our understanding of the evolutionary history of this species. *Migonemyia migonei* may be a species complex [46]. We expect that the present descriptions may contribute to the taxonomic status of *Mg*. *migonei*, at least with respect to the Ceará population which occurs in an area of Brazil that is endemic for cutaneous leishmaniasis.
