# Correction: Morphological aspects of immature stages of *Migonemyia migonei* (Diptera: Psychodidae, Phlebotominae), an important vector of Leishmaniosis in South America, described by scanning electron microscopy

**DOI:** 10.1371/journal.pone.0315234

**Published:** 2024-12-04

**Authors:** Eric Fabrício Marialva, Nágila F. Secundino, Fernando F. Fernandes, Helena R. C. Araújo, Claudia M. Ríos-Velásquez, Paulo F. P. Pimenta, Felipe A. C. Pessoa

The following information is missing from the Funding statement: This work was funded by the Fundação de Amparo à Pesquisa do Estado do Amazonas (FAPEAM; EDITAL N. 005/2019—PAPAC) (FAPEAM; EDITAL N. 005/2019—PAPAC awarded to EFM.
